# Changes in Social Asymmetry as People Age and the Effects of Personality and Socioeconomic Status

**DOI:** 10.1093/geroni/igaf122.280

**Published:** 2025-12-31

**Authors:** Jing Luo, Kathryn Jackson, Emorie Beck, Tomiko Yoneda, Karina Van Bogart, Daniel Mroczek, Anthony Ong, Eileen Graham

**Affiliations:** Northwestern University, Evanston, Illinois, United States; Northwestern University, Evanston, Illinois, United States; University of California, Davis, Davis, California, United States; University of California, Davis, Davis, California, United States; Northwestern University, Evanston, Illinois, United States; Northwestern University, Evanston, Illinois, United States; Cornell University, Ithaca, New York, United States; Northwestern University, Evanston, Illinois, United States

## Abstract

Social stress related experiences are substantial public health concerns as people age. However, prior research suggests discrepancies among different aspects of social stress such as social isolation and loneliness. Social asymmetry is a concept that captures the discordance/concordance between loneliness and social isolation levels. Social asymmetry indicates how well individuals adapt to their social environment, with individuals experiencing greater loneliness than expected given their social isolation levels considered socially vulnerable and those experiencing less loneliness than expected considered socially resilient. It remains unknown how social asymmetry changes over time as people age, and how different individual and contextual factors contribute to individual differences in the change trajectory of social asymmetry. In the current project, we used data from 9 large longitudinal studies to examine changes in social asymmetry during aging and the effects of personality traits and socioeconomic status on the levels of and changes in social asymmetry. Adopting the coordinated data analysis framework allows us to synthesize the results across samples and examine the degree to which the findings are generalizable by testing different sample-level moderators. The preliminary results suggested increases in social asymmetry in late adulthood. Higher neuroticism was found to be related to higher levels of and increases in social asymmetry (social vulnerability, whereas higher extraversion, conscientiousness, and agreeableness, as well as higher SES, were related to lower levels of and decreases in social asymmetry (social resilience). The study enhances our understanding of individual differences in the dynamics of objective and subjective social stress during aging.

